# Systemic inflammatory challenges compromise survival after experimental stroke via augmenting brain inflammation, blood- brain barrier damage and brain oedema independently of infarct size

**DOI:** 10.1186/1742-2094-8-164

**Published:** 2011-11-24

**Authors:** Ádám Dénes, Szilamér Ferenczi, Krisztina J Kovács

**Affiliations:** 1 Laboratory of Molecular Neuroendocrinology, Institute of Experimental Medicine, Budapest, Hungary; 2 Faculty of Life Sciences, University of Manchester, Manchester, M13 9PT, UK

**Keywords:** cerebral ischaemia, blood-brain barrier, oedema, IL-1α, inflammation, systemic, LPS, anaphylaxis

## Abstract

**Background:**

Systemic inflammation impairs outcome in stroke patients and experimental animals via mechanisms which are poorly understood. Circulating inflammatory mediators can activate cerebrovascular endothelium or glial cells in the brain and impact on ischaemic brain injury. One of the most serious early clinical complications of cerebral ischaemia is brain oedema, which compromises survival in the first 24-48 h. It is not understood whether systemic inflammatory challenges impair outcome after stroke by increasing brain injury only or whether they have direct effects on brain oedema, cerebrovascular inflammation and blood-brain barrier damage.

**Methods:**

We used two different systemic inflammatory stimuli, acute endotoxin treatment and anaphylaxis to study mechanisms of brain injury after middle cerebral artery occlusion (MCAo). Ischaemic brain injury, blood-brain barrier damage and oedema were analysed by histological techniques. Systemic cytokine responses and inflammatory changes in the brain were analysed by cytometric bead array, immunofluorescence, *in situ *hibridization and quantitative real-time PCR.

**Results:**

Systemic inflammatory challenges profoundly impaired survival in the first 24 h after experimental stroke in mice, independently of an increase in infarct size. Systemic lipopolysaccharide (LPS) dose-dependently increased mortality (50-100%) minutes to hours after cerebral ischaemia. Acute anaphylactic challenge in ovalbumin-sensitised mice affected stroke more seriously when induced via intraperitoneal administration compared to intravenous. Both LPS and anaphylaxis induced inflammatory changes in the blood and in the brain prior to experimental stroke. Plasma cytokine levels were significantly higher after LPS, while increased IL-10 levels were seen after anaphylaxis. After MCAo, both LPS and anaphylaxis increased microglial interleukin-1α (IL-1α) expression and blood-brain barrier breakdown. LPS caused marked granulocyte recruitment throughout the ipsilateral hemisphere. To investigate whether reduction of ischaemic damage can improve outcome in systemic inflammation, controlled hypothermia was performed. Hypothermia reduced infarct size in all treatment groups and moderately improved survival, but failed to reduce excess oedema formation after anaphylaxis and LPS-induced neuroinflammation.

**Conclusions:**

Our results suggest that systemic inflammatory conditions induce cerebrovascular inflammation via diverse mechanisms. Increased brain inflammation, blood-brain barrier injury and brain oedema formation can be major contributors to impaired outcome in mice after experimental stroke with systemic inflammatory stimuli, independently of infarct size.

## Background

Considerable research supports a relationship between systemic inflammation and poor outcome in stroke patients and in models of experimental stroke [1,2]. Animal models of co-morbidities in stroke have revealed various systemic inflammatory mechanisms which contribute to brain damage. These include peripheral immune cells, proteases, cytokines and chemokines, which can increase ischaemia-induced vascular permeability, excitotoxicity and brain oedema resulting in impaired blood flow recovery, leading to augmented neuronal loss [3,4]. There is a correlation between the size of ischaemic brain injury and the level of central and peripheral inflammatory changes in experimental animals, but this may not be translated easily to stroke patients. It is partially due to the large variability of co-morbidities, age, gender, time of admission after the event and exposure to a wide array of different medicines, which make the influence of systemic inflammation on outcome difficult to assess. In patients acute infection, usually respiratory and of bacterial origin and particularly in the week preceding stroke, is a significant risk factor for cerebral infarction [1]. Inflammatory processes other than infection are also associated with worse otucome after stroke. For example, activation of mast cells, a key cell type in allergy and anaphylaxis is also linked to increased mortality and brain oedema in stroke [5,6].

The treatment of patients with large hemispheric ischaemic stroke accompanied by massive space-occupying oedema represents one of the major unsolved problems in neurocritical care medicine [7]. Some clinical data indicate that neuroprotective approaches may not be sufficient to prevent brain oedema in cerebral ischaemia. For example, hypothermia, a promising treatment for ischaemic stroke due to its neuroprotective effect, was associated with an increase in intracranial pressure and early mortality in a high percentage of patients [7]. In experimental animals, increase in infarct size mostly parallels increased brain oedema and blood-brain barrier (BBB) breakdown, which correlates with worse outcome. However, stroke patients display large variability in recovery depending on location of the infarct and differences in apparent comorbidities [8,9]. This indicates that there is a need to understand the mechanisms how clinically relevant inflammatory events influence outcome after stroke. Therefore we asked whether brain oedema, BBB injury and inflammation are affected similarly by different systemic inflammatory challenges and whether reducing the size of the ischaemic brain damage by hypothermia can lead to proportional reduction of oedema and BBB damage during acute systemic inflammatory conditions. Using two independent peripheral inflammatory stimuli, endotoxin (LPS) and anaphylaxis, in acute cerebral ischaemia in mice we noticed that these conditions can be associated with increased BBB breakdown, inflammation and brain oedema, without significantly affecting infarct size. Moreover, infarct, but not brain oedema was significantly reduced by hypothermia after anaphylactic challenge. Our data indicate that circulating inflammatory markers in different acute systemic inflammatory conditions may worsen outcome after focal cerebral ischaemia by directly increasing BBB damage and brain oedema formation, with a possible involvement of mediators other than most commonly measured cytokines.

## Methods

### Animals

Male, 8-14 weeks old C57BL/6 mice, weighing 25-30 g were used for the study (n = 109). Mice were bred in house, had free access to water and food and were maintained under temperature (21°C ± 1°C), humidity (65%) and light-controlled conditions (12-h light/12-h dark cycle, with lights on at 0700 hours). All animal procedures were carried out in accordance with the European Communities Council Directive (86/609 EEC) and Hungarian Government directive 243/98. Experiments were approved by the Institutional Animal Care and Use Committee at the Institute of Experimental Medicine.

### Systemic inflammatory challenge with Lipopolysaccharide (LPS), ovalbumin sensitisation (OVA) and anaphylaxis (OVA+A) prior to MCAo

Lipopolysaccharide (LPS, serotype: 0111:B4, Sigma L4391) was administered intraperitoneally at doses of 100 μg/mouse (4 mg/kg) 25 μg/mouse (1 mg/kg), 10 μg/mouse (400 μg/kg) and 5 μg/mouse (200 μg/kg). The highest doses (25-100 μg/mouse) were selected to induce substantial systemic inflammation, without compromising survival (LD_50 _value for LPS in mice after intraperitoneal administration is 10-20 mg/kg [10,11]). No animals died or needed to be terminated as a result of LPS injection alone in our experiments. A separate group of mice were sensitised intraperitoneally with 100 μg ovalbumin (Sigma A5378, dissolved in 200 μl saline containing 1 mg Al(OH)_3_). Anaphylaxis was induced 14 days later via injection of 1 mg ovalbumin intraperitoneally (ip.) or intravenously (iv.). Mice were subjected to MCAo 3 h after LPS or anaphylactic challenge.

### Transient focal cerebral ischaemia

Middle cerebral artery occlusion (MCAo) was performed using the intraluminal filament technique as described previously [12]. Anaesthesia was induced with 2% halothane and maintained in 1% halothane-air mixture. For normothermic experiments, core temperature was monitored with a rectal probe and maintained at 37 ± 0.5°C, using a homeothermic blanket during the surgery. For controlled hypothermia, unheated, anaesthetised mice were allowed to spontaneously lose temperature during the surgery until reaching 33 ± 0.5°C and then body temperature was maintained between 33-34°C during occlusion. MCAo was performed with a nylon filament (tip diameter 180 μm, silicone coated), which was introduced into the origin of the external carotid artery and advanced through the internal carotid artery to occlude the MCA. After 60 minutes of occlusion, reperfusion was induced and both normothermic and hypothermic mice were kept at 27°C for 4 h before returning to normal housing. Sham surgery was performed exactly the same as MCAo, but the filament was immediately withdrawn after reaching the origin of the MCA. Mice were subcutaneously injected with 1 mL of sterile saline after the surgery and continuously monitored for neurologic symptoms. Animals were euthanised if they were unable to move spontaneously after 3 h recovery period, or if breathing was seriously compromised. Otherwise, mice were terminated after 24 h reperfusion. Due to the mild to moderate sickness behaviour seen after systemic LPS and anaphylaxis, induction of ischaemia blinded to treatment groups was not fully possible.

### Histology

Mice were anaesthetised and perfused transcardially with 10 mL saline followed by 40 mL 4% paraformaldehyde in 0.1 M Phosphate buffer (pH = 7.4). After cryoprotection of brains in 20% sucrose-KPBS for 24 h, five alternate sets of 20 μm coronal brain sections were cut on a sledge microtome. All sections were collected in an anti-freeze solution (30% ethylene glycol and 20% glycerol in phosphate-buffered saline) and stored at -20°C until processing.

### Measurement of infarct volume and BBB damage

The volume of ischaemic damage was measured using a modification of a method described previously [12]. Briefly, areas of ischaemic damage were identified on cresyl-violet-stained sections at eight neuroanatomically defined coronal levels. Digitized images were created and the areas of damage measured using ImageJ software (NIH, Bethesda, MD, USA). The volume of damage was calculated by integration of areas of damage with the distance between coronal levels. The end points for integration were 2.9 mm (rostral limit) and -4.9 mm (caudal limit) relative to bregma. Volumes are expressed as a percentage of the total hemispheric volume. Leakage of plasma derived IgG into the brain parenchyma was detected by biotinylated horse anti-mouse IgG (Vector Laboratories, BA-2000, 1:500) following blocking nonspecific binding in 5% normal horse serum and 1% BSA. After 30 min incubation with ABC solution (1:500, Vector Laboratories) the reaction was developed with 3,3'-diaminobenzidine (DAB). The volume of BBB damage was calculated as described above. Brain oedema was measured on cresyl violet-stained brain sections and was expressed as a percentage increase of the volume of the ipsilateral hemisphere compared to the contralateral side.

### Immunofluorescence

Free-floating brain sections were blocked with 2% normal donkey serum and incubated overnight with primary antibodies: goat anti-IL-1α (R&D Systems, AF-400-NA) and rabbit anti-granulocyte serum (SJC, kindly provided by Drs. Daniel Anthony and Sandra Campbell, University of Oxford). Then, donkey anti-goat Alexa 488 and donkey anti-rabbit Alexa 594 fluorescent secondary antibodies were used. Sections were stained with biotinylated tomato lectin, which was visualised with streptavidin Alexa 350 conjugate.

### In Situ Hybridization Histochemistry

To monitor IL-1β mRNA, a riboprobe complementary to 373 to 940 nucleotides of the mouse IL-1β gene was transcribed from plasmid (S. Ferenczi) in the presence of ^35^S-UTP. Tissue sections were mounted onto SuperFrost Ultra Plus (Menzer-Glazer) slides post-fixed with 4% paraformaldehyde, digested with Proteinase K (10 in 50 mmol/L Tris, pH = 8 and 5 mmol/L EDTA at 37°C, 5 mins), acetylated (0.25% acetic anhydride in 0.1 mol/L triethanolamine, pH = 8), and dehydrated. Hybridization mixture (50% formamide, 0.3 mol/L NaCl, 10 mmol/L Tris (pH = 8), 2 mmol/L EDTA, 1X Denhardt's, 10% dextran sulfate, 0.5 mg/mL yeast tRNA) was pipetted onto the slides (100 mL, containing probe at 10^7 ^d.p.m./mL) and hybridized overnight at 56°C. Sections were then rinsed in 4X SSC (1X SSC: 0.15 mol/L NaCl and 15 mmol/L trisodiumcitrate buffer, pH = 7), digested with ribonuclease A (20 mg/mL in Tris-EDTA buffer with 0.5 mol/L NaCl at 37°C for 30 min), gradually desalted, and washed in 0.1X SSC at 65 to 75°C for 30 min. Slides were dipped in NTB nuclear emulsion (Kodak) and exposed for 2 weeks, developed in D-19 developer, and lightly counterstained with cresyl violet.

### Quantitative Real-Time PCR

Total RNA was isolated from brain samples with QIAGEN RNeasy Mini Kit (Qiagen, Valencia, CA, USA) according the manufacturer's instruction. To eliminate genomic DNA contamination DNase I treatment was used (100 μl Rnase-free DNase I (1u DNase) solution (Fermentas) was added. Sample quality control and the quantitative analysis were carried out by NanoDrop (Thermo Scientific). Amplification was not detected in the RT-minus controls. cDNA synthesis was performed with the High Capacity cDNA Reverse Transcription Kit (Applied Biosystems, Foster City, CA, USA). The designed primers (Invitrogen, Table 1) were used in the Real-Time PCR reaction with Fast EvaGreen qPCR Master Mix (Biotium, CA, USA) on ABI StepOnePlus instrument. Gene expression was analyzed by ABI StepOne 2.1 program. The amplicon was tested by Melt Curve Analysis on ABI StepOnePlus instrument. GAPDH and ß-actin were used as endogenous control reference genes. Experiments were normalized to GAPDH expression.

**Table 1:** Primers used for real-time PCR

	FORWARD	REVERSE	R^2^	EFF (%)
**GAPDH**	TGACGTGCCGCCTGGAGAAA	AGTGTAGCCCAAGATGCCCTTCAG	0,996	91,7

**ACTIN-β**	CGTAAAGACCTCTATGCCAA	GCGCAAGTTAGGTTTTGTC	0,988	92,5

**IL-1β**	GCCTCGTGCTGTCGGACCCA	TGAGGCCCAAGGCCACAGGT	0,953	98,1

**IL-1α**	CCATAACCCATGATCTGGAAGAG	GCTTCATCAGTTTGTATCTCAAATCAC	0,998	104,0

**IL-17α**	CCTGGCGGCTACAGTGAAG	GGAAGTCCTTGGCCTCAGTGT	0,959	95,8

**IL-6**	CAGTTGCCTTCTTGGGACTGA	GGGAGTGGTATCCTCTGTGAAGTCT	0,959	108,0

**IL-10**	AGTGAGAAGCTGAAGACCCTCAGG	TTCATGGCCTTGTAGACACCTTGGT	0,970	107,2

**G-CSF**	TGCCCAGAGGCGCATGAAGC	GGGGAACGGCCTCTCGTCCT	0,997	102,5

**MCP-1**	CCAGCACCAGCACCAGCCAA	TGGATGCTCCAGCCGGCAAC	0,967	94,8

**TNFα**	CAGCCGATGGGTTGTACCTT	GGCAGCCTTGTCCCTTGA	0,985	102,4

**KC**	GAGCTGCGCTGTCAGTGCCT	CAAGGCAAGCCTCGCGACCA	0,997	105,2

Primers used for real-time PCR (comparative CT experiments) were designed by the Primer Express 3.0 program. Oligonucleotide primer sequences, R2 (regression coefficient) and efficiency (eff %) of the PCR reactions are listed for all primer pairs.

### Measurement of plasma IL-1β and OVA-specific IgE by ELISA

Blood was taken from the right heart ventricle immediately before transcardial perfusion. 3.8% sodium citrate (1:10) was used as an anticoagulant. IL-1β was measured by using mouse IL-1 beta/IL-1F2 DuoSet ELISA kit (DY401, R&D Systems) according to the manufacturers protocol. For the measurement of OVA-specific IgE, Nunc-Immuno^™ ^plates (Sigma) were coated with plasma samples in bicarbonate coating buffer (0.1 M NaHCO_3_, 0.1 M NaCl, pH 8.2) and then blocked with 1% BSA in PBS. Biotinylated anti-mouse IgE antibody was used to detect plasma OVA-specific IgE and mouse anti-ovalbumin IgE (Serotec, MCA2259) antibody was used as standard. The reaction was developed with SAV-HRP (R&D Systems) followed by OptEIA™ TMB Substrate Reagent Set (BD Biosciences).

### Cytokine measurements with cytometric bead array (CBA)

Plasma samples were analysed for 11 key inflammatory cytokines (G-CSF, IFNγ, IL-1β, IL-1α, IL-10, IL-17, IL-6, KC, MCP-1, TNFα, RANTES) by using appropriate CBA Flex Sets (BD Biosciences) according to the manufacturer's protocol. To measure the same cytokines in brain homogenates, animals were transcardially perfused with saline 3 hours after systemic inflammatory challenges. Brain hemispheres were homogenised as described elsewhere [13].

### Data analysis

Quantitative analysis was done in a blinded manner whenever it was possible. Data were analysed by using One-way or Two-way analysis of variance (ANOVA) followed by Bonferroni's post hoc multiple- or paired comparison. Data are expressed as mean ± SEM.

## Results

### Systemic inflammation impairs survival after experimental stroke

**Figure 1:**
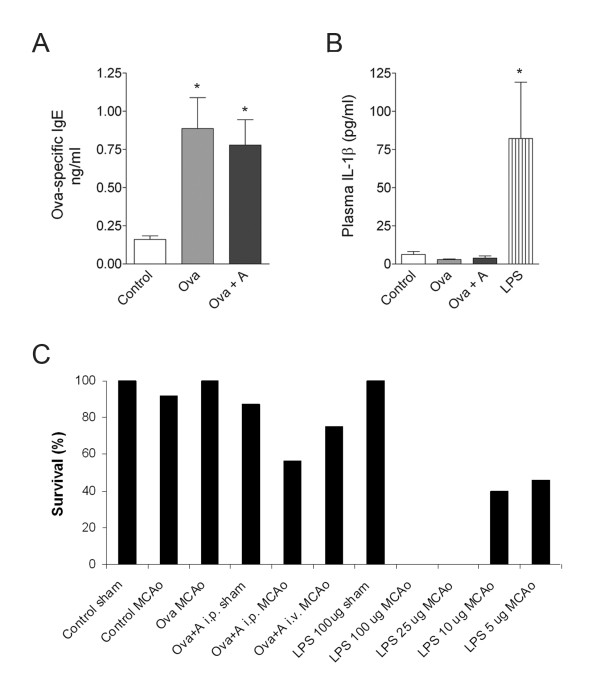
**Systemic inflammatory challenges compromise survival after experimental stroke**. **A**. Measurement of Ova-specific IgGE in plasma with ELISA confirms proper sensitization to antigen in Ova injected mice without (Ova) and with (Ova+A) anaplylaxis. **B**. Plasma IL-1β levels were determined with ELISA after 60 min MCAo and 24 h reperfusion. **C**. Percentage values show the average survival rate of mice at 24 h reperfusion after various challenges and MCAo or sham surgery. Anaphylaxis induced by intraperitoneal (i.p.), intravenous (i.v.) Ova administration in sensitized mice. Systemic inflammation induced by i.p. LPS injection 3 hours prior to MCAo profoundly increase mortality within 24 hours (n = 4-11/group). One-way ANOVA followed by Bonferroni's comparison (*P < 0.05 vs Control).

Mice were challenged with LPS or Ova+A 3 h before MCAo to develop a systemic inflammatory response [14-17] by the time experimental stroke was induced. Mice injected with LPS (25-100 μg/mouse) displayed mild to moderate sickness behaviour, respectively, which was less pronounced at doses of 5-10 μg. Ova sensitised mice showed signs of ear swelling and itching after anaphylactic challenge, which largely resolved after 1-2 h. Successful sensitisation against Ova was confirmed by elevated circulating Ova-specific IgE levels (Figure 1A). Plasma IL-1β was upregulated in LPS-treated animals (Figure 1B), indicating the development of a systemic inflammatory response. Only actively moving animals which showed no signs of serious illness 3 h after LPS or Ova+A challenge were subjected to 60 min MCAo.

Mice challenged intraperitoneally with Ova+A showed impaired recovery after MCAo and only 56% of mice survived for 24 h (Figure 1C). However, 75% of the animals survived if mice were challenged with intravenous Ova indicating that Ova is likely to be acting locally on sensitised peritoneal cells to initiate a systemic inflammatory response, compromising survival after experimental stroke. Mice sensitised with Ova without anaphylaxis expressed no mortality during these studies after MCAo.

No mice survived after MCAo if they received 25-100 μg LPS. Very poor recovery and high mortality (50-60%) was observed with 5-10 μg LPS after MCAo, whereas even high doses of LPS did not compromise survival after sham surgery (Figure 1C). These data showed that circulating inflammatory mediators induced by two completely different stimuli can profoundly impair survival after experimental stroke.

### Systemic inflammatory challenges augment cerebrovascular inflammation, BBB damage and brain oedema after experimental stroke, independently of infarct size

**Figure 2:**
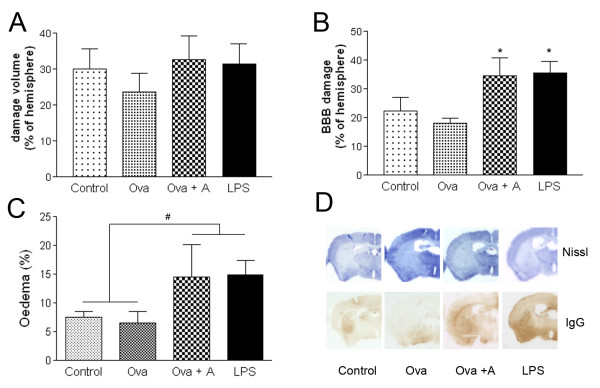
**Systemic inflammation increases BBB damage and brain oedema independently of infarct size**. **A**. The volume of the ischaemic damage was measured on cresyl violet (Nissl) stained serial brain sections. **B**. BBB damage was assessed by immunostaining for mouse IgG on free floating brain sections. Intraperitoneal LPS injection and anaphylaxis 3 hours prior to MCAo significantly augment BBB disruption at 24 hours of reperfusion (P < 0.05, One-way ANOVA, Bonferroni's post-hoc comparison). **C**. Oedema is expressed as percentage increase of the ipsilateral hemispheric volume compared to the contralateral hemisphere (# P < 0.05, systemic inflammatory challenges versus control conditions, Two-way ANOVA). **D**. Representative images show the ischaemic brain- (Nissl) or BBB (IgG) damage in different treatment groups. Data are representative of 4-5 mice/group.

**Figure 3:**
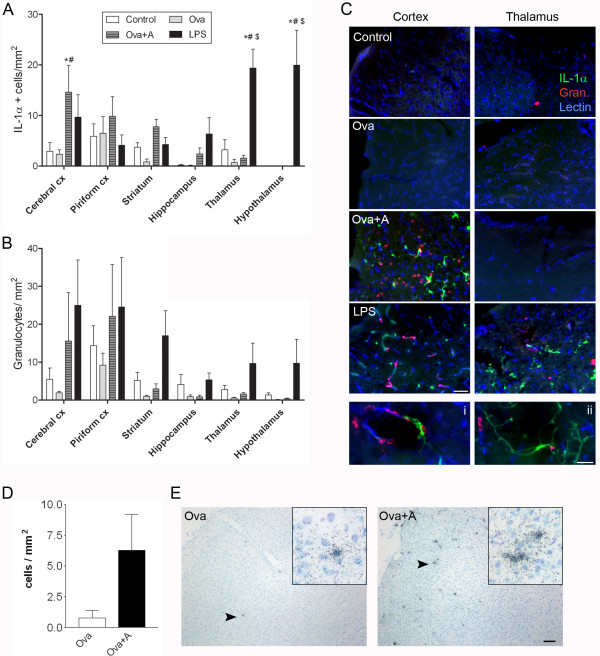
**Experimental stroke-induced IL-1 expression is increased in the brain after LPS administration and anaphylaxis**. Quantification of IL-1α-positive cells (**A**) and granulocytes (**B**) in the ipsilateral hemisphere. **C**. Immunofluorescence shows IL-1α-positive cells (green), granulocytes (red) and tomato lectin-positive blood vessels (blue) and microglia/macrophage cells in the ipsilateral cerebral cortex and thalamus after 24 h reperfusion. Perivascular cells in the cerebral cortex (i) and parenchymal cells in the thalamus (ii) expressing IL-1α after MCAo are abundant in mice with anaphylaxis and LPS treatment, respectively. **D**. *In situ *hybridization reveals increased number of cells expressing IL-1β mRNA in the ischaemic cerebral cortex of mice 24 h after MCAo with anaphylaxis (Ova+A) compared to MCAo and Ova sensitization (Ova). **E**. Silver grains indicate the presence of IL-1 β mRNA in Ova and Ova+A mice after MCAo (counterstained with cresyl violet). Inserts show higher magnification of IL-1β mRNA containing cells indicated by arrowheads. Data are representative of 4-5 mice in each group. One-way ANOVA followed by Bonferroni's comparison (P < 0.05 vs * Control, # Ova, $ Ova+A). Scale bars: **C**ii - 20 μm, **C **- 50 μm, **E **- 100 μm.

Mice that survived for 24 h were investigated to reveal how different systemic stimuli affected brain damage caused by focal cerebral ischaemia. No significant increase of ischaemic brain damage was observed after LPS or Ova+A challenges (Figure 2A). However, both conditions increased BBB damage (Figure 2B). Systemic inflammation resulted in increased brain oedema (systemic inflammatory challenges versus control conditions, P < 0.05, Two-way ANOVA). Sensitisation with Ova without anaphylaxis did not have any detrimental effect on oedema or BBB damage (Figure 2B-D). Next, we examined whether different systemic inflammatory conditions had an effect on brain inflammation after MCAo. Anaphylaxis significantly increased the expression of IL-1α in the ipsilateral cerebral cortex (Figure 3A, C), while endotoxin-induced systemic response was associated with profound increases of IL-1α in remote areas, such as the thalamus and the hypothalamus (P < 0.05). In addition to parenchymal cells, several IL-1α-positive cells were observed perivascularly around dilated blood vessels after anaphylaxis, indicating that IL-1α expression may be associated with the development of vasogenic oedema (Figure 3C, i). In contrast, LPS-treated mice displayed mostly parenchymal, elongated, IL-1α-positive cells with microglial morphology (Figure 3C, ii). IL-1α expression was detected mostly in tomato lectin-positive ramified microglia/macrophage cells (not shown). LPS treatment resulted in an overall increase of granulocytes in the ipsilateral hemisphere (P < 0.05), whereas this was only observed in the cerebral cortex after anaphylaxis (Figure 3B, C).

We also measured IL-1β expression in the brain after MCAo, using *in situ *hybridization. IL-1β has been associated with brain oedema formation after stroke [4,18-20]. We did not find significant changes in IL-1β mRNA expression after MCAo in LPS-treated mice (data not shown) compared to controls, but observed markedly increased numbers of IL-1β mRNA expressing cells after anaphylaxis (Figure 3D, E). In line with the IL-1α data, IL-1β mRNA expression was augmented mostly in the ischaemic cerebral cortex (Figure 3B, C) at 24 h reperfusion, whereas we only occasionally observed IL-1β-positive cells in the ipsilateral striatum (data not shown).

### LPS and anaphylaxis-induced systemic inflammatory responses are dominated by different circulating cytokines

**Figure 4:**
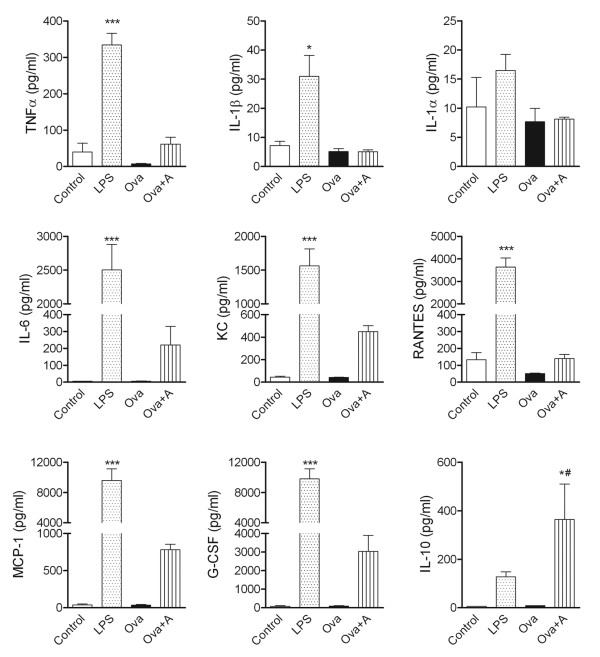
**Cytokine responses in the plasma after systemic inflammatory challenges**. Cytokines in plasma samples were measured by cytometric bead array. Mean ± SEM values are shown, n = 3-7. One-way ANOVA followed by Bonferroni's comparison (P < 0.05 vs * Control, # Ova).

To investigate whether these fundamentally different systemic inflammatory stimuli drive different systemic inflammatory responses, we investigated the plasma levels of 11 key cytokines 3 h after LPS or anaphylaxis (at the time MCAo is performed). LPS induced profound (10-1000 fold) increases in G-CSF, IL-6, KC, MCP-1, TNFα, and RANTES and a 4-fold, but significant increase in IL-1β, without significantly affecting IFNγ, IL-17A, IL-1α and IL-10 levels (Figure 4). Anaphylaxis increased G-CSF, IL-6, KC, MCP-1, TNFα, and RANTES levels 4-30 fold (not significant), and significantly elevated IL-10 levels (80 fold) while no IL-1β or IL-1α was detected (Figure 4).

### Systemic inflammatory challenges induce neuroinflammatory changes in the brain

**Figure 5:**
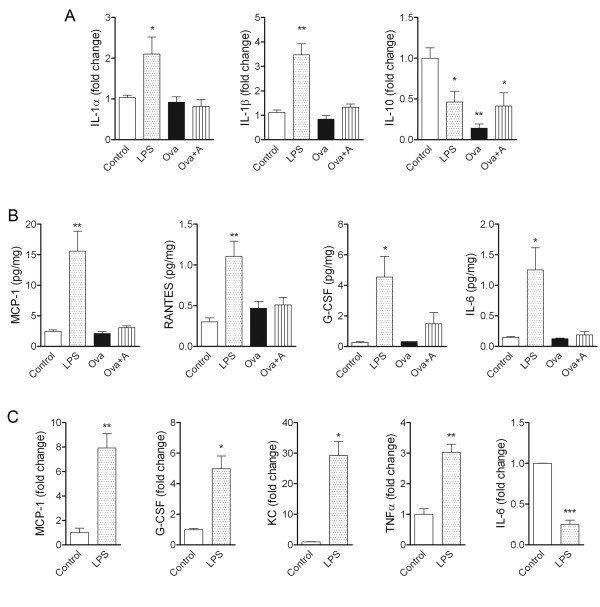
**Systemic inflammatory challenges induce neuroinflammatory changes in the brain**. **A**. In homogenates of brain hemispheres, real-time PCR reveals changes in cytokine expression 3 h after intraperitoneal LPS administration or anaphylaxis. Values represent fold changes over control. **B**. Cytometric bead array shows increased cytokine levels in the brain 3 hours after LPS treatment. **C**. Elevated cytokine mRNA levels confirm central induction of inflammation after peripheral LPS treatment. Cytokine expression was analysed with real-time PCR in brain hemisphere samples. Data are representative of 3-7 mice in each group. One-way ANOVA followed by Bonferroni's post-hoc comparison (*P < 0.05, **P < 0.01).

In order to examine inflammatory changes in the brain in response to systemic inflammatory challenges, we measured central IL-1α, IL-1β and IL-10 expression with real-time PCR, 3 h after LPS treatment or anaphylaxis. In the absence of any direct brain injury, intraperitoneal LPS caused a 2-3 fold increase in central IL-1α and IL-1β mRNA levels in brain hemishpheres compared to control mice (Figure 5A). In contrast, IL-10 mRNA levels were decreased in LPS and Ova treated mice and after anaphylaxis compared to controls (Figure 5A).

Cytometric bead array did not show significant elevation in IL-1α, IL-1β or IL-10 protein concentration in the brain after LPS administration or anaphylaxis (data not shown), but revealed a significant increase in MCP-1 (6 fold), RANTES (3-4 fold), G-CSF (17 fold) and IL-6 (9 fold) levels in response to LPS (Figure 5B). KC (CXCL-1) was elevated in the brain 2-40 fold after LPS treatment, which was not significant due to large variations among individual animals (data not shown).

Real-time PCR confirmed the induction of MCP-1, G-CSF, and KC in the brain after intraperitoneal LPS treatment and also revealed higher levels of TNFα mRNA compared to control mice (Figure 5C). Interestingly, IL-6 mRNA levels were significantly reduced after LPS administration (Figure 5C).

### Hypothermia profoundly reduces ischaemic brain injury, but does not prevent the effect of systemic inflammation on brain oedema and survival

**Figure 6:**
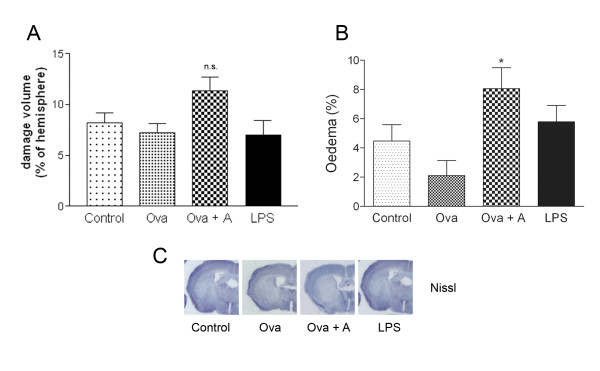
**Hypothermia reduces brain injury but does not prevent increased brain oedema formation after anaphylaxis**. Hypothermia combined with 60 minutes of MCAo results in profound protection against ischaemia in all animal groups compared to normothermia (P < 0.001, see Figure 2). Body temperature was reduced to 33°C by spontaneous heat loss during surgery and was maintained between 33-34°C during occlusion. **A**. The volume of the ischaemic damage was measured on cresyl violet (Nissl) stained serial brain sections (**B**). **C**. Anaphylaxis significantly increases brain oedema after MCAo and 24 hours of reperfusion compared to Ova sensitization (P < 0.05, One-way ANOVA, Bonferroni's post-hoc comparison) in hypothermic mice with 60 minutes of MCAo. Data are representative of 5 mice/group. n.s.- not significant.

To further investigate the mechanisms of how peripheral inflammatory challenges affect ischaemic brain damage and inflammation, we subjected mice to hypothermia during occlusion, using the same experimental groups as above. Overall, hypothermia profoundly reduced ischaemic brain damage (P < 0.001) and brain oedema (P < 0.01) compared to normothermia (Figure 6A, B). Only LPS-treated mice showed elevated granulocyte levels and IL-1α expressing cells in the ipsilateral hemisphere after hypothermia and MCAo (not shown), indicating that peripheral LPS initiates brain inflammation even if the ischaemic brain damage is reduced. Hypothermia was not sufficient to protect against increased brain oedema caused by anaphylaxis compared to control mice (Figure 6C). Moreover, hypothermia only moderately reduced mortality in mice exposed to LPS or anaphylaxis (72% survival after LPS and 67% survival after anaphylaxis).

## Discussion

Here we present experimental evidence that two different systemic inflammatory conditions, endotoxin treatment and anaphylaxis markedly compromise survival, augment brain oedema, BBB damage and cerebrovascular inflammation after experimental cerebral ischaemia. We also show that this effect is not a direct consequence of increased ischaemic brain damage in the current model, and reduction of the infarct by hypothermia cannot fully prevent increased mortality (after both LPS and anaphylaxis) and oedema (after anaphylaxis). The key message of our study is that a number of complications associated with systemic inflammation in stroke patients may be in part caused by an elevated inflammatory burden and not only consequences of a large infarct in response to pre-existing inflammation or infection.

Our aim was to investigate the effect of two acute systemic inflammatory conditions, which involve fundamentally different mechanisms of induction, on brain injury and survival. LPS stimulates toll-like receptor 4 (TLR4), which results in the release of key proinflammatory cytokines from various cell types [21]. Elevated levels of IL-1β, TNFα and IL-6 have been reported in the circulation, while IL-6 can also be detected in the cerebrospinal fluid (CSF) within 2-5 h after intaperitoneal LPS administration [15,22]. We found high levels of circulating inflammatory cytokines and elevated IL-1β levels 3 h after LPS challenge, the time when MCAo was induced. It is likely that activation of TLR4 by host-derived ligands can also impact on stroke outcome as TLR4-deficient mice are protected against the ischaemic injury [23]. The classic pathway of systemic anaphylaxis is induced largely by activation of mast cells and results in the release of various vasoactive and inflammatory mediators, such as histamine and platelet-activating factor [24]. In our study, successful activation of Ova-sensitized mast cells was also indicated by elevated levels of IL-10 [25]. Mast cells are early responders to stroke and are involved in the regulation of acute BBB changes after cerebral ischaemia [6]. Inhibition of mast cell function reduces brain oedema formation after intracerebral haemorrhage [26]. Therefore, our data show that the induction of systemic inflammation was successful with two independent models, which are both relevant to ischaemic stroke.

It is generally difficult to evaluate the exact mechanisms whereby systemic inflammation affects ischaemic brain injury in experimental studies, because larger strokes are mostly associated with elevated levels of inflammation in the brain. The key finding of our study was the profound effect of systemic inflammatory challenges on survival and brain inflammation after stroke, which took place independently of the increase in infarct size. Moreover, both conditions (LPS and anaphylaxis) increased BBB damage compared to control animals, which is likely to contribute to the formation of brain oedema. Peripherally administered LPS has minimal penetration across the murine blood-brain barrier [27] but LPS and circulating inflammatory mediators can induce central inflammatory actions through receptors expressed in the circumventricular organs [28,29]. Our data show that central inflammatory responses have been altered due to systemic LPS challenge and anaphylaxis prior to the induction of experimental stroke. Increased BBB damage, brain oedema and the induction of IL-1α in the hypothalamus, thalamus and in perivascular macrophages/microglia after MCAo in response to LPS might be associated with direct effects of LPS on circumventricular organs or signals mediated by perivascular receptors of LPS and/or other inflammatory mediators. A previous study showed low mortality, but increased ischaemic- and BBB damage after MCAo and intraperitoneal LPS administration compared to MCAo alone [18]. However, in that study the time of LPS administration was 30 min, and not 3 h prior to MCAo as in our model, which may result in different systemic levels of proinflammatory mediators by the time MCAo is performed. In contrast, intraperitoneal IL-1β administration was found to increase mortality by 60% [18], which is strikingly similar to what we observed after LPS challenge.

Analysis of central cytokine changes indicated an altered inflammatory status in the brain after systemic inflammatory challenges. The induction of IL-1α, IL-1β, TNFα MCP-1, RANTES, G-CSF, KC mRNA and increased MCP-1, RANTES, G-CSF protein levels in the brain after systemic LPS challenge are likely to result in a primed proinflammatory response by the time experimental stroke is induced. This, together with altered IL-10 mRNA levels after both anaphylaxis and LPS may partially explain elevated microglia-derived IL-1, increased brain oedema and BBB damage seen after MCAo. The downregulation of IL-6 mRNA and the increase in IL-6 protein levels 3 h after LPS administration are controversial and may indicate a complex and time-dependent regulation of central inflammatory responses at mRNA and peptide levels by systemic challenges.

To our knowledge, no studies have investigated the effect of anaphylaxis on experimental stroke so far. We show that anaphylaxis profoundly impaired survival in the same time frame as LPS treatment did, in spite of the fundamentally different mechanisms of induction as outlined above. After anaphylaxis, we found upregulation of several proinflammatory cytokines, although high levels of circulating IL-10 were measured. IL-10 protects against brain injury [30,31], which may explain why we did not observe an increase in infarct volume after MCAo in these mice. In contrast, high numbers of IL-1α-immunopositive and IL-1β mRNA expressing cells and dilated blood vessels were seen in the brain after anaphylaxis and MCAo, which indicates that these changes were initiated in response to systemic inflammatory challenge. Anaphylaxis may exert its effects on brain inflammation through other inflammatory mediators than the cytokines examined. Although LPS increased proinflammatory cytokines several fold compared to anaphylactic challenge and initiated a more pronounced granulocyte response, IL-1α-positive cells after endotoxin treatment were dominantly expressed in remote areas. However, our data show that both LPS and anaphylaxis result in the release of various vasoactive substances into the circulation, which can alter vascular permeability and therefore lead to oedema in central and peripheral tissues [32-35].

One possible explanation for not observing increased infarct volume in these studies may be the loss of the most serious cases prior to 24 h reperfusion, which could result in increased numbers of mice with smaller infarct after systemic inflammatory challenge. However, in our hands the current MCAo model did not result in maximal cortical damage in any of the treatment groups (Figure 2), which argues against this explanation. To further examine mechanisms whereby systemic inflammatory challenges affect brain injury, we also repeated MCAo and all treatments under hypothermic conditions. Although we found significant protection with respect to ischaemic brain damage by hypothermia in all groups, mice that underwent anaphylaxis and MCAo displayed increased brain oedema compared to controls. Hypothermia has been shown to exert neuroprotection and can markedly reduce infarct size in experimental rodent models [36,37]. However, our data indicate that at least in part, brain oedema may be directly affected by circulating inflammatory mediators and is not only a consequence of increased brain damage after stroke with systemic anaphylaxis. These results indicate the need for precise understanding of the effect of systemic inflammatory changes on oedema formation after stroke. Systemic inflammation is not only a predictor to worse outcome in stroke patients [2,3] but anaphylactoid reactions may occur in response to thrombolytic treatment in stroke patients taking an angiotensin-converting-enzyme inhibitor [38,39]. Cerebral ischaemia can also occur in patients after wasp sting anaphylaxis [40,41] indicating that changes in blood coagulation may be involved in this process.

Taken together, these data demonstrate the involvement of acute systemic inflammation in increased mortality, BBB damage and brain oedema formation after experimental stroke. The fact that these changes are not neccessarily linked to increased ischaemic brain damage indicate that appropriate management of stroke patient with various comorbidities in the future may require precise understanding of the interaction between peripheral inflammatory processes and cerebral ischaemia.

## Competing interests

The authors declare that they have no competing interests.

## Authors' contributions

AD and KJK designed the studies. AD performed experiments, AD and SF performed measurements and analysed the data. AD and KJK wrote the paper. All authors read and approved the final manuscript.
